# Supplementary material for: Sex and gender considerations in implementation interventions to promote shared decision making: A secondary analysis of a Cochrane systematic review
Source: PLoS One. 2020 Oct 8;15(10):e0240371. doi: 10.1371/journal.pone.0240371 (PMC7544054; doi:10.1371/journal.pone.0240371)
Supplement: S1 Table — (DOCX) [file pone.0240371.s002.docx]

**S1 Table**: Description of the criteria according to the studies characteristics (Sensitivity analyses)

|  | | Use of categories to describe Sex  (n=58) | | | | | Use of categories to describe Gender  (n=58) | | | | | | Non-interchangeable use of Sex and Gender (n=83) | | | |
| --- | --- | --- | --- | --- | --- | --- | --- | --- | --- | --- | --- | --- | --- | --- | --- | --- |
| **Characteristics** | **Appropriate or Unclear** | | **Inappropriate** | | | ***P-value*** | | **Appropriate**  **or Unclear** | | **Inappropriate** | | ***P-value*** | **Yes** | **No or Unclear** | | ***P-value*** |
| **Years of publication** | | |  | | *.999^(c)^* | |  | |  | | *.347^(a)^* | |  |  | *.750^(a)^* | |
| <2016 | | 35 (85.4) | 6 | |  | | 21 (51.2) | | 20 | |  | | 28 (43.1) | 37 |  | |
| ≥2016 | | 15 (88.2) | 2 | |  | | 11 (64.7) | | 6 | |  | | 7 (38.9) | 11 |  | |
| **Countries in which**  **studies were conducted** | | | | *.627^(a)^* | | |  | |  | | *.444^(a)^* | |  |  | *.991^(a)^* | |
| North America | | 27 (90.0) | 3 | |  | | 18 (60.0) | | 12 | |  | | 19 (42.2) | 26 |  | |
| Europe/other | | 23 (82.1) | 5 | |  | | 14 (50.0) | | 14 | |  | | 16 (42.1) | 22 |  | |
| **Interventions targeting** | | |  | | *.641^(a)^* | |  | |  | | *.332^(a)^* | |  |  | *.592^(a)^* | |
| Patients only | | 20 (83.3) | 4 | |  | | 16 (66.7) | | 8 | |  | | 20 (46.5) | 23 |  | |
| Health  Profesionals only | | 9 (81.8) | 2 | |  | | 5 (45.5) | | 6 | |  | | 4 (30.8) | 9 |  | |
| Both | | 21 (91.3) | 2 | |  | | 11 (47.8) | | 12 | |  | | 11 (40.7) | 16 |  | |
| **Effect of interventions**  **on primary outcome** | | | | | *.670^(a)^* | |  | |  | | *.061^(a)^* | |  |  | *.386^(a)^* | |
| Significant | | 7 (43.8) | 9 | |  | | 12 (75.0) | | 4 | |  | | 11 (50.0) | 11 |  | |
| Non-significant/  Data not reported | | 21 (50.0) | 2 | |  | | 20 (47.6) | | 22 | |  | | 24 (39.3) | 37 |  | |

(a) = Pearson Chi-squared test; (b) = Yates’s Khi-2 Correction for continuity; (c) = Fischer exact test
